# Tumor dormancy: potential therapeutic target in tumor recurrence and metastasis prevention

**DOI:** 10.1186/2162-3619-2-29

**Published:** 2013-10-16

**Authors:** Sih-han Wang, Shiaw-Yih Lin

**Affiliations:** 1 Department of Systems Biology, Unit 950, The University of Texas MD Anderson Cancer Center, 1515 Holcombe Blvd., Houston, TX 77054, USA

**Keywords:** Tumor dormancy, Quiescence, Immunologic dormancy, Angiogenic dormancy, Tumor microenvironment

## Abstract

In past decades, cancer patient survival has been improved with earlier detection and advancements in therapy. However, many patients who exhibit no clinical symptoms after frontline therapy subsequently suffer, often many years later, aggressive tumor recurrence. Cancer recurrence represents a critical clinical challenge in effectively treating malignancies and for patients’ quality of life. Tumor cell dormancy may help to explain treatment resistance and recurrence or metastatic reactivation. Understanding the dormant stage of tumor cells may help in discovering ways to maintain the dormant state or permanently eliminate dormant residual disseminated tumor cells. Over the past decade, numerous studies indicate that various mechanisms of tumor dormancy exist, including cellular dormancy (quiescence), angiogenic dormancy, and immunologic dormancy. In this short review, we summarize recent experimental and clinical evidence for these three mechanisms and other possible tumor microenvironment mechanisms that may influence tumor dormancy.

## Introduction

Tumor dormancy is a recognized clinical phenomenon in which disseminated tumor cells (DTCs) remain occult, asymptomatic, and undetectable over a prolonged period of time. Dormancy can occur at the earliest stage of tumor development but also when remnant tumor cells escape treatment. Tumor dormancy may contribute to tumor progression and relapse, either locally or metastatically at distant sites, years or decades after treatment. Clinical dormancy is frequently observed in many types of tumors, such as breast cancer (BC) [1], prostate cancer (PC) [2,3], melanoma [4,5], and B-cell lymphoma [6,7]. Dormant cells are characterized by their slow growth, their ability to escape frontline treatment and host immunity, and their capability to self-renew. A clinical study showed that non-proliferating cancer cells could persist during chemotherapy and were detected more frequently in BC patients with progressive disease than in patients with primary BC [8].

**Figure 1:**
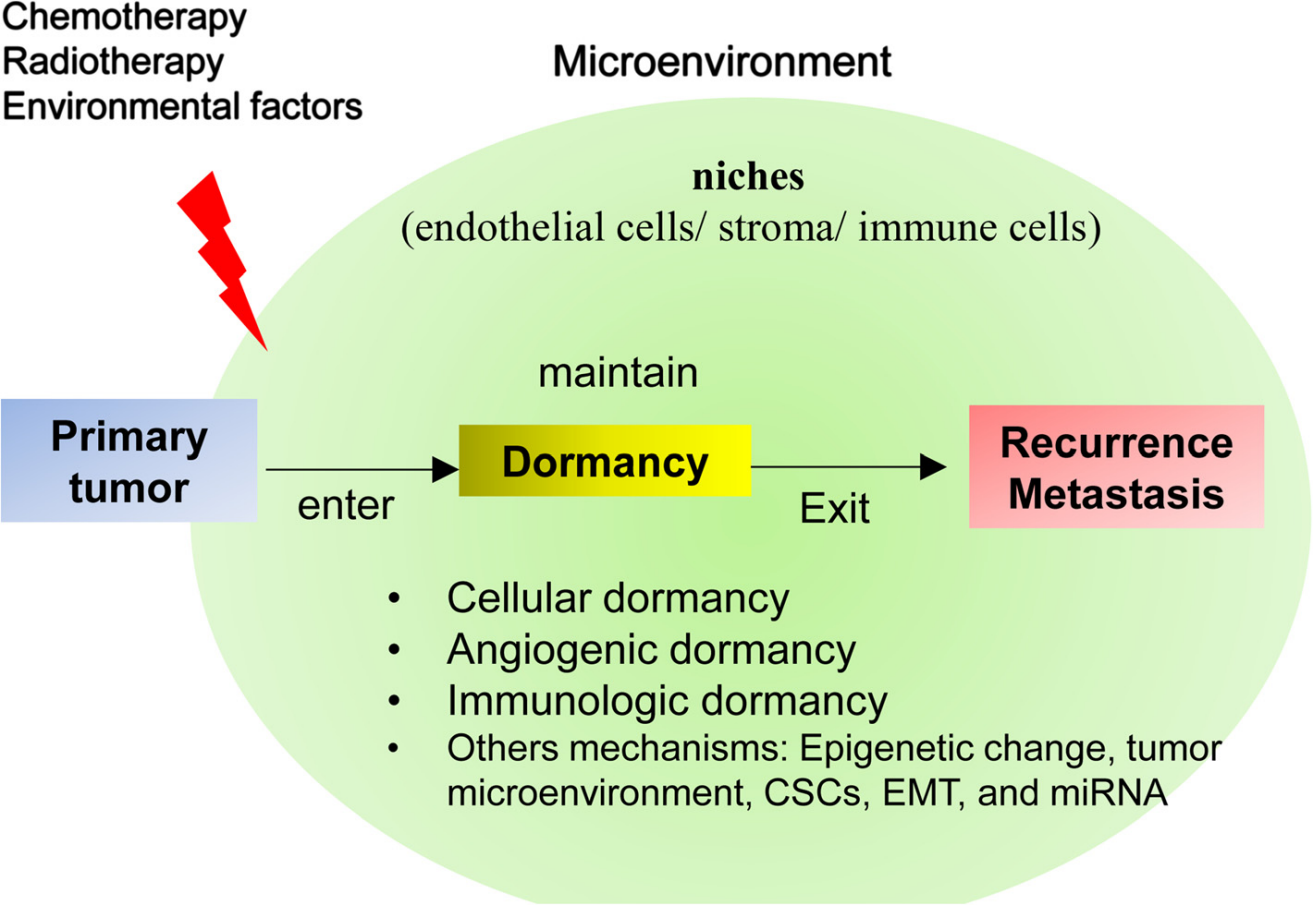
**Mechanisms in tumor dormancy.** Tumor dormancy can lead to tumor recurrence locally or to metastasis at a distant site. Dormancy can be induced by more than one mechanism: cellular dormancy (quiescence), angiogenic dormancy (tumor mass size limit), and immunologic dormancy (immunosurveillance, balance between proliferation and apoptosis). Others mechanisms, such as epigenetic change, tumor microenvironment, CSCs, EMT, and miRNA, also may involve in tumor dormancy. A tumor microenvironment that is altered (such as by frontline treatment) can mediate tumor cell entrance into, maintenance, and exit from dormancy through interaction with cells at niches, such as endothelial cells, stroma, or immune cells.

Three molecular mechanisms involved in tumor dormancy have been identified: cellular dormancy (quiescence or mitotic arrest), angiogenic dormancy (limited tumor size), and immunologic dormancy (immunosurveillance, balance between clearance and proliferation) (Figure 1). The mechanisms that regulate the transition between the dormant and proliferating states are largely unknown. In addition, no dormancy markers have been well characterized. Here, we review recent findings of relevant genetic abnormalities, novel discoveries regarding tumor dormancy, and possible mechanistic links. This information should help in the development of appropriate study models and improve therapeutic approaches to prevent disease recurrence.

### Cellular dormancy

Cellular dormancy is a state in which cells are in a quiescent state (the G0 phase). These cells do not enter the normal proliferating cell cycle. Their growth arrest is reversible and under certain conditions, such as induction by growth factors, cytokines, nutrients, or chemical agents, the cells can re-enter the cell cycle to proliferate again. Quiescent normal adult stem cells serve as a source of self-renewal in the maintenance of multiple adult epithelial tissues. In contrast, quiescence in a heterogeneous cancer population likely contributes to cancer cell survival in response to anti-cancer agents and radiotherapy, leading to disease recurrence [9-11].

In patients with prolonged clinical dormancy, the presence of dormant cells is often identified by their lack of the cellular proliferation marker Ki-67 as well as the lack of apoptotic markers [12-14]. Cellular DNA/RNA content analysis is commonly used to assess for cells in the G0 phase, as indicated by 2 N DNA ploidy and reduced RNA content. In contrast, cells in the G1/S/G2 phase exhibit 2 N/4 N DNA and higher RNA content [15].

**Figure 2:**
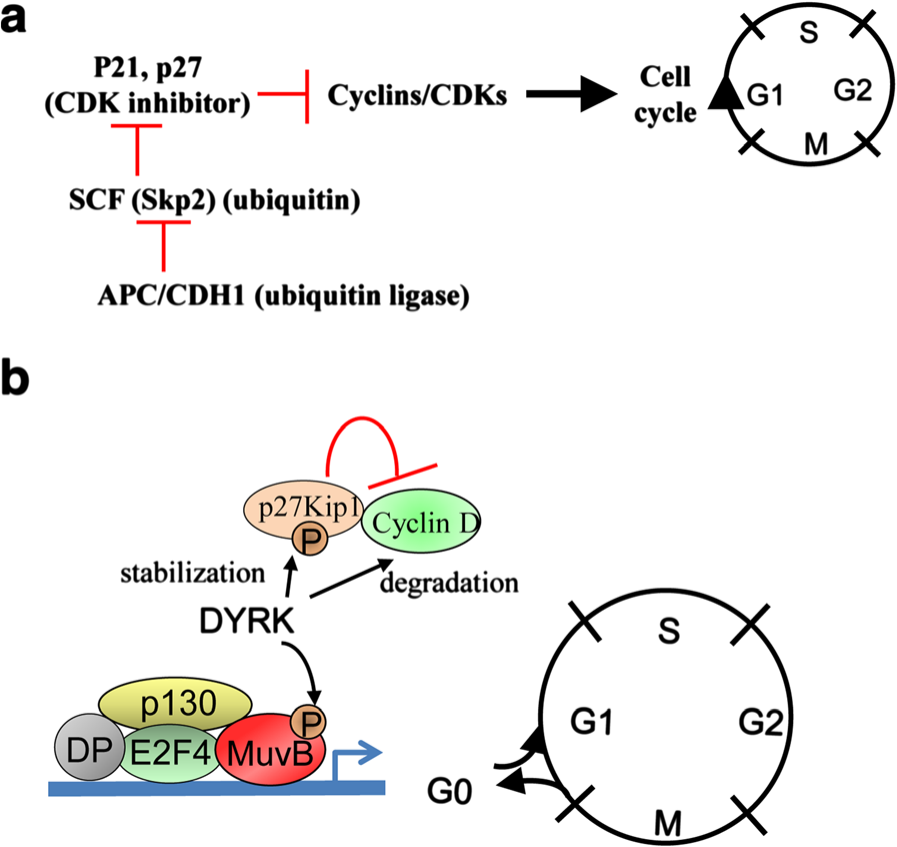
**Role of cell cycle regulators in the cellular dormancy (“intrinsic dormancy”) mechanism. (a)** In the “typical” cell cycle pathway, upregulation of CDK inhibitors, such as p21 and p27, can inhibit cells from entering the cell cycle by downregulating cyclin/CDK activation. In this scenario, cells can enter into a quiescent state (G0). The ubiquitin ligase APC/CDH1 enhances the upregulation of p21/p27 expression by increasing the degradation of Skp2 (one of the units that constitute SCF, a p21/p27/p57 inhibitor). **(b)** The DREAM complex in the quiescent state (G0). MuvB recruits and interacts with p130 (RB-like), E2F4, and dimerization partner (DP), forming the DREAM complex. DYRK phosphorylates MuvB and activates the DREAM complex, which triggers cells to become quiescent. DYRK also stabilizes p27Kip1 through phosphorylation and prevents cells from moving into the G1 phase by reducing the expression of cyclin D.

Several cell cycle regulators are known to directly or indirectly mediate cell quiescence. The cyclin-dependent kinase (CDK) inhibitors p27Kip1 and p21 can cooperate to maintain a balance between hematopoietic stem cell dormancy and proliferation at certain cell-cell interactions at tumor microenvironmental niches [16]. For example, cell adhesion of lymphoma cells to bone marrow (BM) stromal cells was shown to lead to reversible cell cycle arrest through upregulation of posttranscriptional levels of p27Kip1 and p21 through activation of the APC/CDH1 ubiquitin ligase complex [17]. In addition, APC/CDH1 increased the ubiquitination and degradation of Skp2 (part of the p21 and p27 inhibitor, SCF) and subsequently reduced the level and ubiquitination and degradation of p27Kip1, which resulted in cell cycle arrest (Figure 2a).

Besides the APC/CDH1-SKP-p27Kip1/p21 signaling pathway, the multi-subunit DREAM complex was recently indicated as a critical regulator of quiescence. This complex consists of a dimerization partner, a Retinoblastoma (RB)-like pocket protein (p130, encoded by RB-like 2 or p107, encoded by RB-like 1), E2F protein, and mutil-vulval class B protein (MuvB). The MuvB core component is essential for coordinating cell cycle; it recruits, binds, and directs key transcription factors to the promoters of periodic cell cycle genes during various phases of the cell cycle [18]. During quiescence and with a high expression level of p130, MuvB binds to p130, E2F, and the dimerization partner to form the DREAM complex and repress all cell cycle-dependent expression [18-20] (Figure 2b). Impairing various components of the DREAM complex by mutation or RNA interference leads to disruption of cell cycle-dependent repression of genes, such as *p107/p130* and *E2F4/E2F5*, during quiescence. The expression level of p107 is high only in proliferating cells, but knockdown of p130 results in increased expression of p107, which results in the formation of a p107-DREAM complex in quiescent cells. However, p130^-/-^/p107^-/-^ mouse embryonic fibroblasts lose the ability to form a functional DREAM complex and fail to repress E2F-dependent genes expression during quiescence, causing cells to re-enter the cell cycle [21]. E2F4^-/-^/E2F5^-/-^ mouse embryonic fibroblasts can also re-enter the cell cycle from quiescence but fail to arrest in the G1 phase when the CDK inhibitor INK4A is overexpressed [22].

Dual specificity tyrosine phosphorylation-regulated kinase (DYRK), a serine/threonine kinase, maintains quiescence by activating the DREAM complex. DYRK1A and DYRK1B contribute to the phosphorylation of the MuvB subunit LIN52 at serine 28, which promotes interaction between the MuvB core and p130-E2F during quiescence [23]. Knockdown or disruption of DYRK1A activity leads to reduced levels of MuvB phosphorylation and assembly of the DREAM complex [23]. DYRK1B can stabilize p27Kip1 [24] and increase the turnover of cyclin D isoforms [25], thereby preventing cells from entering the cell cycle and thus maintaining their quiescence (Figure 2b).

In summary, a balance between quiescent DREAM complex and proliferative complexes, the MuvB-BMYB-FOXM1 complex and the typical RB-cyclin/CDK-dependent complex, is critical to maintain quiescence. Modulating functions of the DREAM complex in synchrony with anti-proliferative treatments may provide a novel therapeutic approach to primary cancer treatment and relapse prevention.

### Angiogenic dormancy

**Figure 3:**
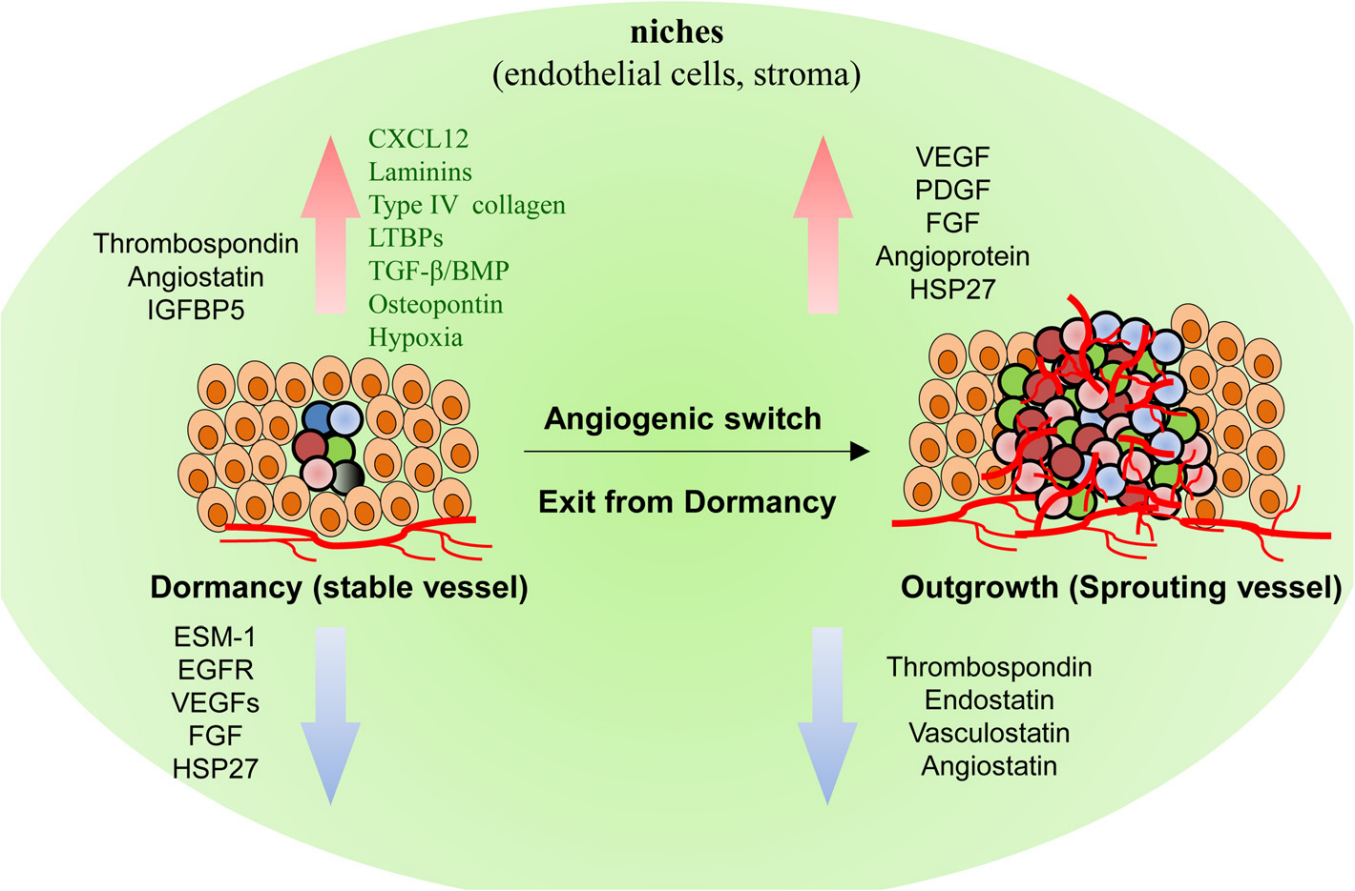
**Angiogenic dormancy (“extrinsic dormancy”) at niches.** At niches, dormancy can be induced through signaling between DTCs, endothelial cells, and stroma with both upregulation and downregulation of multiple factors and signaling axes. DTCs enter dormancy with a vascular structure that is stable and non-angiogenic. In contrast, a sprouting vascular structure triggers tumor cells to exit from dormancy following an “angiogenic switch” in microenvironment, leading to tumor outgrowth and recurrence or metastasis. BMP, bone morphogenetic protein; CXCL12, Chemokine (C-X-C motif) ligand 12; EGFR, epithelial growth factor receptors; ESM-1, endothelial specific marker 1; FGF, fibroblast growth factor; HSP27, heat shock protein 27; IGFBP5, insulin-like growth factor binding protein 5; LTBPs, latent transforming growth factor β binding protein; PDGF, platelet-derived growth factor; TGF-β, transforming growth factor β; VEGFs, vascular endothelial growth factors.

Once DTCs exit from quiescence and proliferate to reach a certain tumor mass size that requires more than the normal tissue vasculature can provide, the tumor mass induces angiogenesis to meet its nutrient demands. However, some tumor cells lack this angiogenic capacity and are therefore unable to grow beyond a certain size. These cells are controlled by a balance between cellular proliferation and apoptosis and remain clinically undetectable, resulting in angiogenic dormancy. The transition from a pre-vascular tumor to a highly vascularized and progressively growing tumor is known as the “angiogenic switch” [26]. The balance between pro-angiogenic factors (such as vascular endothelial growth factor (VEGF), platelet-derived growth factor, fibroblast growth factor, and angiopoietin) and anti-angiogenic factors (such as thrombospondin (TSP), endostatin, vasculostatin, and angiostatin) plays a role in the angiogenic switch of dormant cells (Figure 3).

A recent xenograft model study with U-87 human glioblastoma cells in SCID mice indicated that the balance between pro-angiogenic and anti-angiogenic factors is associated with the dormancy or aggressiveness of tumor growth [27]. The “aggressive” clones of U-87 cells formed larger and vascularized tumor masses, whereas the “dormant” clones formed smaller and avascular tumor masses. Compared with aggressive clone tumor cells, these dormant clones expressed high levels of TSP, angiomotin, and insulin-like growth factor binding protein 5 and low levels of endothelial cell-specific marker 1 and epithelial growth factor receptor. In addition, the dormant clone tumor cells exhibited impaired angiogenesis and slower invasion.

In a BC xenograft study, heat shock protein 27 (HSP27) was identified as a key player in the balance between tumor dormancy and outgrowth with the onset of angiogenesis [28]. Reduced HSP27 protein expression in angiogenic cancer cells led to a low secretion level of pro-angiogenic factors (such as VEGF-A, VEGF-C, and basic fibroblast growth factor), which resulted in poorer proliferation and migration of cancer cells and effectively suppressed tumor growth and angiogenesis. In contrast, overexpression of HSP27 in non-angiogenic cancer cells promoted tumor growth *in vivo*. For BC or melanoma patients, a low expression level of HSP27 protein was associated with less aggressive tumors and improved patient survival [28].

Chemokine signaling directs certain tumor cells to specific organs that provide a supportive microenvironment with cytokines or chemokines, tumor-derived secreted factors, and tumor-specific homing-regulated receptors through cross-talk among metastatic tumor cells, stroma, and organ-derived cells. This environment determines whether DTCs survive, proliferate, become dormant, or undergo apoptotic cell death. This niche may be not required for recurrence or metastasis, but they do promote recurrent and metastatic progression, and support tumor cell colonization.

Gap junctional intercellular communication between BC cells and bone marrow (BM) stroma can facilitate BC dormancy. A subset of BC cells with the properties of dormancy, including chemoresistance, cycling quiescence, asymmetric division, and highly invasive capability, is responsible for gap junctional intercellular communication with BM stroma [29]. Interaction between BM stroma-derived CXCL12 and BC-expressing CXCR4 or CXCR7 receptors can direct BC cells from circulation into BM stromal niches, causing BC cells to become quiescent in the BM stroma [30].

Recently, a unique organotypic microvascular niche model demonstrated that endothelial-derived TSP-1 and endothelial-derived perlecan maintain BC cells in a quiescent state and suppress tumor growth [31,32]. Other extracellular matrix molecules, such as laminin, type IV collagen, and latent transforming growth factor β (TGF-β) binding protein (LTBP), also contribute to dormant niches [33]. That study showed that the sprouting of neovasculature disrupted vascular homeostasis at dormant niches, which led to the loss of suppressive signals. Sequentially, extracellular matrix molecules and growth factors promote micro-metastatic outgrowth of BC cells.

TGF-β/bone morphogenetic protein (BMP) signaling between bone stromal cells and PC cells at bone niches also mediates the balance between the dormancy of PC cells and metastasis [34]. Secreted from bone stromal cells, BMP7 interacts with the BMP receptor 2 of PC cells; this interaction activates p38, p21, and NDRG1, thereby inducing dormancy. In a mouse model, BMP7 injection significantly limited tumor growth, whereas inhibition of BMP7 increased bone metastasis. In addition, in PC patients, upregulation of BMP receptor 2 expression correlated with the extent that bone was free of metastasis.

The anti-adhesion matrix component LTBP-2 is also associated with pre-metastatic niches for dormancy [33]. Low LTBP-2 expression levels were observed in nasopharyngeal carcinoma (NPC) cell lines and tumor samples from NPC patients. Overexpression of LTBP-2 induced dormancy and inhibited NPC cell migration and angiogenesis through the reduction of VEGF, which led to attenuated tumor formation. In contrast, inhibition of TGF-β/BMP signaling, such as by overexpression of Coco (a secreted antagonist of TGF-β ligands), induced the exit of BC cells from dormancy, specifically in the lung niches, leading to metastatic outgrowth [35].

Osteopontin (OPN) is involved in leukemic dormancy in BM niches and in the persistence of minimal residual disease after chemotherapy. During circulation and migration, proliferating acute lymphoblastic leukemia blasts express high levels of OPN receptors, such as VLA-4, and then specifically adhere to stroma-derived OPN, which is secreted by osteoblasts at endosteal niches. Acute lymphoblastic leukemia blasts also secrete OPN into the local microenvironment. With additional niche-specific microenvironmental factors in BM, leukemic blasts return to dormancy, resulting in resistance to chemotherapeutic agents and eventually tumor recurrence. Neutralization of OPN triggers dormant cells to re-enter the cell cycle, sensitizing them to chemotherapeutic agents [36].

In summary, in the tumor microenvironment or pre-metastatic niches, communication between cancer cells and cells at the niches is critical to the regulation of DTC dormancy and to the angiogenic switch with neovascular structure homeostasis. A particular challenge is to develop appropriate clinical models to identify the biomarkers and therapeutic targets in angiogenic-dormant tumor cells. Three-dimensional co-culture systems with tissue-like morphology may provide the best advantage for investigating the mechanisms of angiogenic dormancy in the tumor microenvironment [27].

### Immunologic dormancy

**Figure 4:**
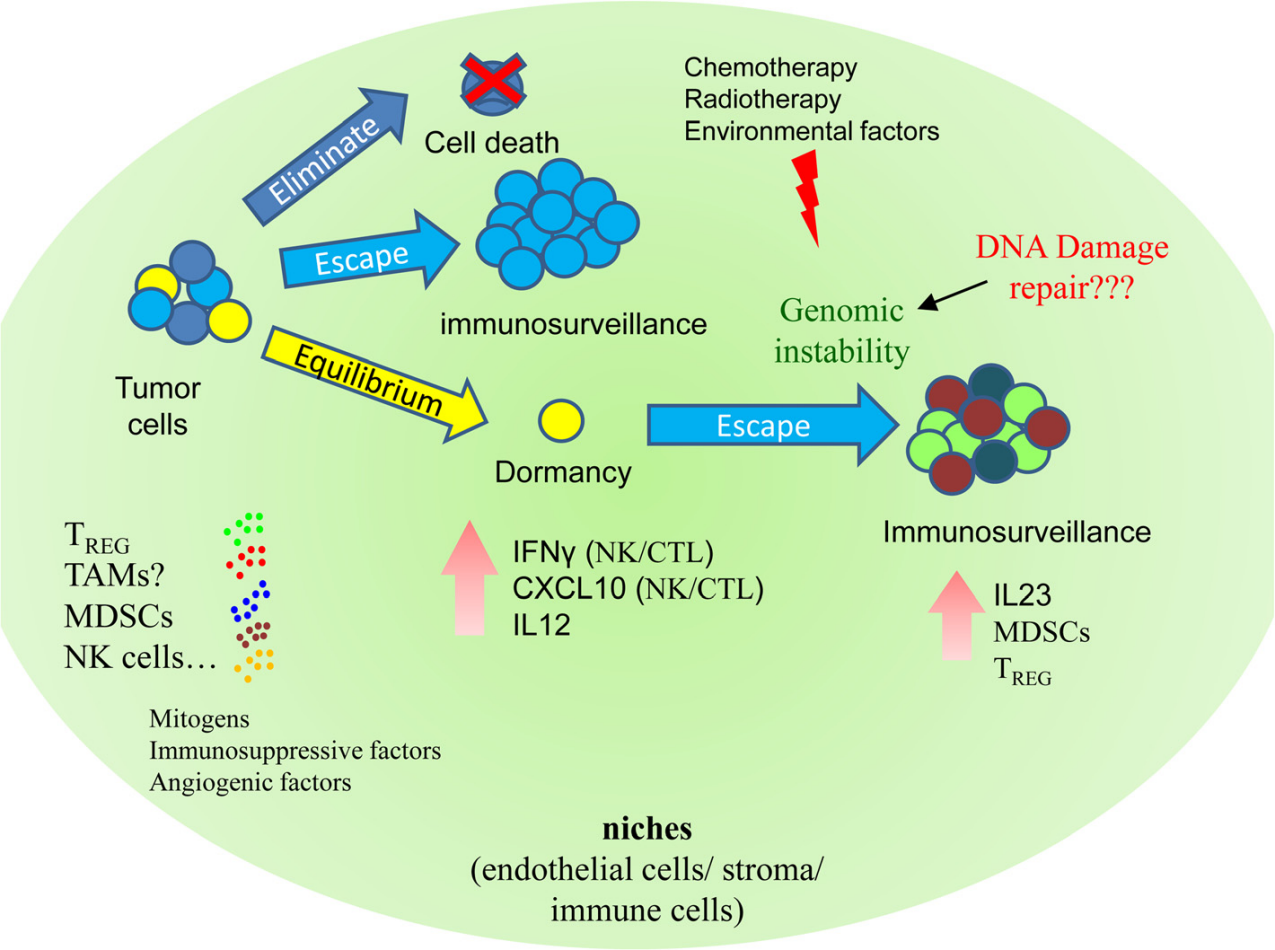
**Immunologic dormancy (“equilibrium dormancy”) at niches.** The immune system can control tumor cells through elimination, equilibrium, or escape. DTCs can enter dormancy during equilibrium states. Over years, environmental factors change or the genomic stability of DCTs changes, allowing dormant cells to escape (this state is called immunosurveillance) and reactivate to grow. At niches, immune cells, stroma, and endothelial cells can secret mitogens, immunosuppressive factors, and angiogenic factors that modify the tumor microenvironment and trigger cells to exit from dormancy and lead to recurrence or metastasis. CTL, cytotoxic T-lymphocytes; CXCL10, C-X-C motif chemokine 10; INFγ, Interferon gamma; MDSCs, myeloid-derived suppressor cells; NK, natural killer; TAMs, tumor-associated macrophages; T_REG_, regulatory T-cells.

Immunity also contributes to tumor dormancy under an equilibrium condition with immunologic clearance. How DTCs enter into, maintain, and exit from this immunologic dormancy is poorly understood. Immunity can control tumor cells through three distinct processes: elimination, equilibrium, and escape (Figure 4). Elimination functions as an extrinsic tumor suppressor in naïve hosts in which innate and adaptive immunity work together to detect and destroy transformed cells before they become clinically symptomatic. Certain DTC variants may not be completely eliminated, but their net growth is restricted by immunity control, resulting in an equilibrium state and maintenance of tumor cells in dormancy. These DTCs may induce a host-protective immune response, or their antigen expression (recognized by adaptive immunity *via* T-cell effectors) may be modified to adapt to the host immune system. Over a prolonged period, the constant interaction of immunity with DTCs may edit the immunogenicity of dormant tumor cells, causing them to enter into an attenuated immunogenic state. This attenuated state leads to the escape process. The edited DTCs begin to grow progressively in an immunosuppressive tumor microenvironment and eventually become clinically detectable and lead to tumor recurrence [37-39].

Direct or indirect modulation of the immune system in the tumor microenvironment can enhance the escape of tumor cells from dormancy. For example, DTCs can directly attenuate the cytotoxic T-lymphocyte response by reducing T-cell activation, promoting DTC resistance to cytotoxic T-lymphocyte-induced apoptosis, or altering tumor-associated antigen presentation to antigen-presenting cells [40,41]. Numerous immune cell types may enhance indirect escape from dormancy through their infiltration into the tumor microenvironment; these cell types include myeloid-derived suppressor cells, regulatory T-cells, and tumor-associated macrophages [27,42,43]. These cells can secret numerous mitogens, cytokines, and some angiogenic factors to promote angiogenesis, cell proliferation, and immunosuppression [40]. For example, interleukins 23 and 12 were shown to play critical and opposing roles in immunologic dormancy in a mouse model of methycholanthrene-induced fibrosarcoma [44]. Interleukin 23 can suppress both innate and adaptive anti-tumor effectors responses, whereas interleukin 12 prevents cancer outgrowth [44,45].

Immunologic dormancy may rely on the high genomic instability of DTCs. Once dormant DTCs accumulate genomic aberrations years or decades after radiotherapy or chemotherapy, genomic instability generates a less immunogenic phenotype that can better evade anti-tumor immunity, resulting in reactivation of these cells. Genome or exome sequencing analysis of unedited and edited DTCs may reveal mutations involved in the process of exit from dormancy as a consequence of genomic instability. Specific mutations in these edited tumor cells may lead to altered expression of tumor-specific antigens for T-cells, and these antigens could serve as immunotherapeutic targets.

### Other potential mechanisms in tumor dormancy

Several other mechanisms may play roles in tumor dormancy, such as genetic and epigenetic changes, cancer stem cells, tumor microenvironment, epithelial-mesenchymal transition, and non-coding RNA manipulation. Epigenetic modification by DNA methyltransferase and histone deacetylase within DTCs may mediate the expression of genes that trigger cells to enter into, maintain, or exit from dormancy. For example, altered tumor-associated antigen expression through epigenetic change in DTCs may lead to the escape of these cells from immunity and reactivate their growth. Epigenetic change also may regulate quiescence through the nontraditional RB/p27 pathway.

CSCs have similar properties as dormant cells, including slow growth, escape frontline treatment and host immunity, and capability to self-renew. However, whether cancer stem cells involve in tumor dormancy is controversial. One group identified a population of slow-cycling BC cells with retained metabolism labeling. These cells exhibited increasing capability of chemotherapy resistance and leading to tumor recurrence in CSC-independent model [46]. In contrast, one subset of BC with CSC marker (Oct4hi/CD44hi/med/CD24-/+) and properties can facilitate dormancy in pre-metastatic niches [29]. Prostate CSCs with senescence reversibility in BMP7-dependent manner contribute dormancy in bone [27].

Stress signaling or suppressive signals in the tumor microenvironment may reverse the epithelial-mesenchymal transition in tumor cells and induce tumor cells into dormancy or quiescence. However, a subsequent angiogenic switch or altered immunity eventually reactivates dormant tumor cells, resulting in tumor recurrence. Tumor microenvironment also facilitates a switch between proliferation and dormancy by the balance between two mitogen-activated protein kinase (MAPK) signaling pathways, the extracellular signal-regulated kinase (ERK) and p38 signaling pathways [47-50]. Angiogenesis-associated mitogen-activated protein kinase (uPAR) functions as a central regulator of the ratio of ERK/p38. Loss of uPAR signaling from tumor microenvironment results in stress signaling with low ERK and high p38 activities, which in turn lead to dormancy. In contrast, tumor microenvironment shift may lead to reactivate uPAR signaling following high ERK and low p38 activities, which can reactivate dormant cells, leading to recurrence and metastasis [51].

Non-coding RNAs were recently identified as potential biomarkers in human cancers. A single microRNA (miRNA) molecule can affect the expression of hundreds of genes and numerous signaling pathways [52,53]. miRNA may affect dormancy through various pathways. For example, miRNA-190 is upregulated in dormancy-prone glioblastomas and osteosarcomas, and it mediates several transcriptional factors, tumor suppressor genes, and interferon response pathways [27]. In summary, epigenetic modification or non-coding RNAs may be able to regulate dormancy. Epigenetic and non-coding RNA microarray techniques could help identify potential dormancy regulators and pathways. However, developing study models and distinguishing between dormant and heterogeneous tumor populations are complicated and challenging tasks.

Kim and colleagues recently published a gene signature for tumor cell dormancy that was based on results from two clinical studies on tumor cell quiescence or angiogenic failure [36]. The 49-gene signature they developed represents a dormancy score, which is based on upregulated and downregulated genes in dormant cells [54]. The researchers applied the dormancy score to human BC samples from four microarray studies and concluded that estrogen receptor-positive tumors had a higher dormancy score, which correlated with a low proportion of metastasis, whereas estrogen receptor-negative tumor had a lower dormancy score, which correlated with a higher proportion of metastasis. Whether this gene signature is actually associated with dormancy and recurrence/metastasis and whether it could serve as a tumor dormancy biomarker need to be confirmed with an appropriate dormancy model.

The DNA damage repair response, especially the pathway that responds to DNA double-strand breaks, may protect dormant tumor cells from radiotherapy or chemotherapy. An example is the non-homologous end joint (NHEJ) pathway. In this pathway, the broken ends are directly ligated without the need for a homologous template, in contrast to homologous recombination. While the homologous recombination pathway is error free, the NHEJ pathway is error prone. As DNA damage accumulates in DTCs following cancer treatment, NHEJ may be upregulated to repair these damages and allow tumor cells to survive; however, this pathway also creates genomic instability in the tumor cells. In certain microenvironments, these genomically unstable DTCs may become dormant, maintain dormancy, or eventually reactivate, becoming more aggressive and leading to cancer recurrence. This idea needs to be tested *in vitro* and *in vivo* with an appropriate dormancy model.

## Conclusion

Several mechanisms and pathways involved in regulating tumor cell dormancy have recently been identified. However, most of these mechanisms are not specific to dormancy. In addition, the lack of suitable model systems for detecting and maintaining the dormant state creates challenges in designing systematic studies on dormancy either *in vitro* or *in vivo*. Continued investigation and discovery are needed to identify dormancy signatures and biomarkers as well as the therapeutic targets and windows for “secondary” cancer prevention after primary treatment has failed. Better understanding of tumor cell dormancy will lead to novel detection techniques in the clinic and therapeutic options to prevent deadly tumor recurrence and metastasis.

## Abbreviations

BC: Breast cancer; BM: Bone marrow; BMP: Bone morphogenetic protein; CDKs: Cyclin-dependent kinases; ERK: Extracellular signal-regulated kinase; DTC: Disseminated tumor cell; DYRK: Dual specificity tyrosine phosphorylation-regulated kinase; HSP27: Heat shock protein 27; LTBP: Latent transforming growth factor β (TGF-β) binding protein; MAPK: Mitogen-activated protein kinase; miRNA: MicroRNA; MuvB: Mutil-vulval class B protein; NHEJ: Non-homologous end joint; NPC: Nasopharyngeal carcinoma; OPN: Osteopontin; PC: Prostate cancer; TGF-β: Transforming growth factor β; TSP: Thrombospondin; VEGF: Vascular endothelial growth factor.

## Competing interests

The authors declare that they have no competing interests.

## Authors’ contributions

SHW drafted and coordinated the manuscript. SHW and SYL read and approved the final manuscript.
